# Complicated jejunal diverticulitis: A case report and review of literature

**DOI:** 10.1002/ccr3.6570

**Published:** 2022-11-15

**Authors:** Daniel Scheese, Yahya Alwatari, Jamal Khan, Ashley Slaughter

**Affiliations:** ^1^ Department of Acute Care Surgery Virginia Commonwealth University Richmond Virginia USA

**Keywords:** diverticulitis, gastroenterology, general surgery, jejunal diverticulitis, small intestine

## Abstract

Jejunal diverticulitis is an uncommon pathology wherein a delay in diagnosis can lead to significant morbidity and mortality. We report a case of such diverticula requiring operative management, after patient failed non‐operative management, likely due to advanced jejunal inflammation from a delay in diagnosis and subsequent management.

## Abstract 2

With a low reported incidence rate, jejunal diverticulitis can be easily overlooked in elderly patients presenting with nausea and abdominal pain. A delay in diagnosis and treatment has potential for intestinal obstruction, bleeding, and perforation. Mortality rates of 40% have been reported in cases of perforation secondary to jejunal diverticulitis.

## 1 INTRODUCTION

With a low annually reported incidence of 0.3%–2.3%, jejunal diverticulitis is rarely seen in a hospital setting, with most cases seen in male patients between 50 and 70 years of age.^1^, ^2^ Although also infrequently encountered, asymptomatic jejunal diverticulosis is more commonly seen, with small bowel outpouchings usually found incidentally on computed tomography (CT) imaging.^2^ The pathophysiology behind jejunoileal diverticulosis is thought to be due to smooth muscle motor dysfunction resulting in disordered contractions. This motor dysfunction causes an increased intraluminal pressure, leading to herniation of the mucosa and submucosa through the mesenteric side of the bowel.^3^ These diverticula have the potential to become inflamed, resulting in jejunal diverticulitis, and cause complications. Most studies report the acute complications of these diverticula to be intestinal obstruction, bleeding, inflammation, and perforation.^1^ In this report, we present a case of delayed diagnosis of jejunal diverticulitis highlighting the diagnostic difficulty, as well as the need to maintain a high level of suspicion regarding future cases.

## 2 MATERIAL AND METHODS

An electronic database (PubMed) was searched by two separate researchers for published studies mapping to Medical Subject Heading (MeSH) terms jejunal diverticulitis, antibiotics, operative management, and nonoperative management. The data from the published articles regarding patient demographics, presentation, length of stay (LOS), and management were compiled and described.

## 3 CASE HISTORY

**FIGURE 1:**
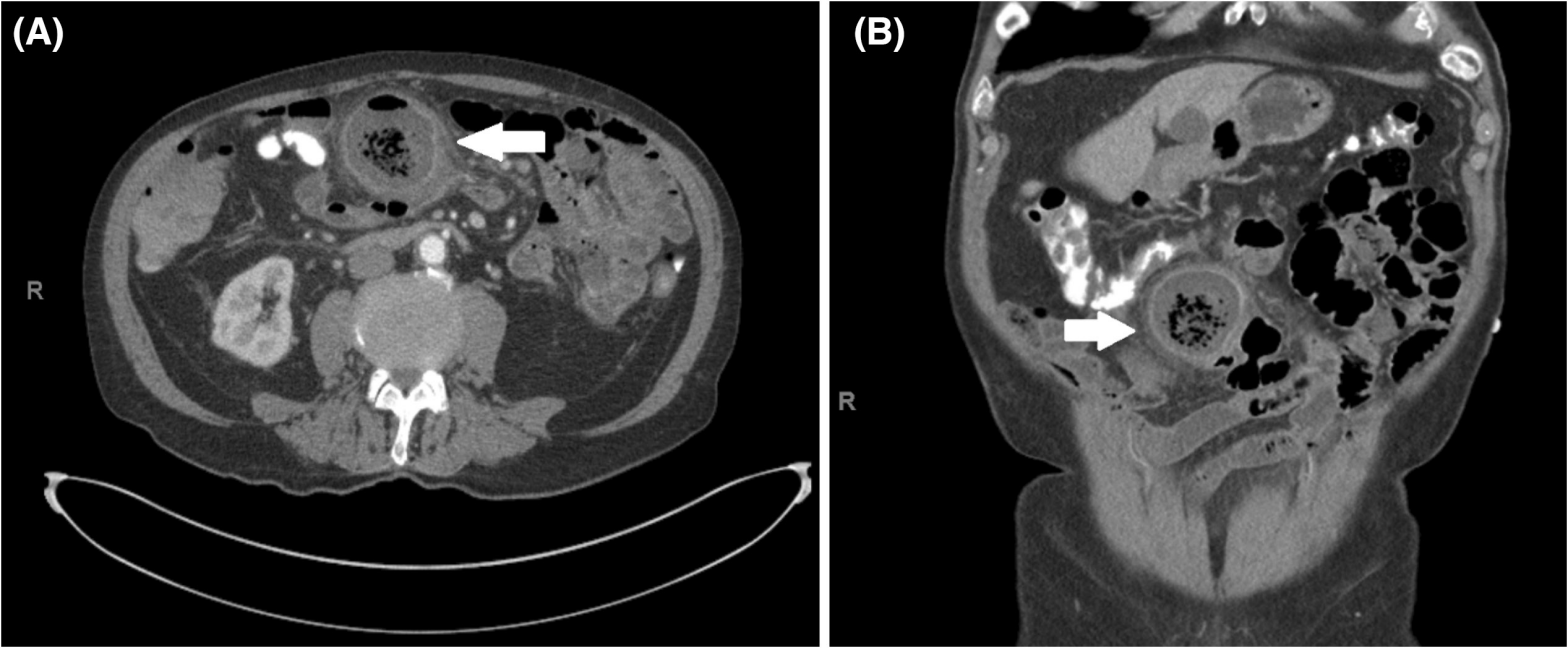
Contrast‐enhanced CT scan of the abdomen/pelvis: (A) axial image. (B) Coronal image. Prominent small bowel diverticulum measuring 5.2 × 5.4 cm with internal fecal contents, with tall thickening and adjacent fat stranding (arrow)

**FIGURE 2:**
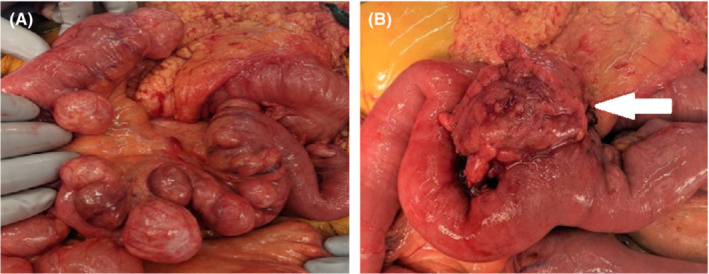
Intraoperative gross images of (A) segment of jejunal diverticuli, and (B) large 3.5 × 3.0 cm inflamed jejunal diverticulum identified on preoperative CT scan (arrow)

An 85‐year‐old man presented as a hospital transfer with leukocytosis, nausea, and worsening intermittent, sharp, periumbilical abdominal pain previously requiring multiple visits to an outside emergency room. The patient had been treated with oral antibiotics as an outpatient for suspected sigmoid diverticulitis but had failed outpatient management. During his third visit to the outside emergency department, a CT scan was obtained which demonstrated proximal small bowel diverticulosis and severe sigmoid diverticulosis, with no diverticulitis noted. He also reported a bright red bowel movement prior to transfer. With the combination of periumbilical abdominal pain, bright red bowel movement, leukocytosis, and severe sigmoid diverticulosis demonstrated on CT scan, colonic diverticulitis was suspected. The patient was transferred to our facility and admitted to the emergency general surgery service, where he was initially treated conservatively with intravenous antibiotics and bowel rest. By hospital Day 4, he remained afebrile and hemodynamically stable but continued to endorse focal tenderness to palpation in the periumbilical region, and a second CT scan was obtained which demonstrated a prominent small bowel diverticulum with internal fecalization and newly seen wall thickening with adjacent fat stranding (Figure 1A,B). Redemonstration of extensive sigmoid diverticulosis was also seen on the repeat CT scan. On hospital Day 5, due to the lack of clinical improvement, the decision was made to proceed with operative intervention addressing the inflamed small bowel diverticula. A midline laparotomy was performed, and the small bowel was run from the ligament of Treitz to the ileocecal valve. The patient was found to have a 60 cm segment of jejunum, beginning 30 cm from the ligament of Treitz, with multiple small and medium diverticula and one large, inflamed diverticulum at the distal end of this segment (Figure 2A,B). Of note, there were no diverticula in the remainder of the small bowel. Given this anatomy, the decision was made to perform a segmental resection of this 60 cm jejunal segment with a primary anastomosis, which was completed without issue. The specimen was sent to pathology as a permanent specimen for gross and microscopic evaluation, which revealed diverticular disease with an associated organizing abscess and fibrosis. The remainder of the patient's course was uncomplicated. He had return of bowel function and tolerated a regular diet by post‐operative day five, at which time he was discharged home.

## 4 DISCUSSION

Jejunal diverticulitis is a rare disorder associated with a high morbidity and mortality, primarily due to the elderly age group of the patients most commonly affected. The aforementioned case demonstrates the symptomatic potential of the disease. Although the reported incidence rate of jejunal diverticulosis is low, some studies have shown 10%–15% of diagnosed cases required surgical intervention for sequelae of jejunal diverticulitis including perforation, obstruction, peritonitis, or gastrointestinal bleeding.^1^ Mortality rates of 40% can be seen in patients with perforation secondary to jejunal diverticulitis.^4^

Due to its uncommon nature, diagnosis can often be delayed or missed. If jejunal diverticulitis is suspected, an abdominal acute series can be obtained with attention to any free air under the diaphragm indicating perforation, or signs of obstruction indicated by dilated loops of bowels with air‐fluid levels. CT scanning can provide additional detail and information regarding the segment of bowel affected, as well as additional lesions, and evidence of inflammation, such as thickening of the bowel wall and fat‐stranding.

The treatment of choice for complicated jejunal diverticulitis is an exploratory laparoscopy or laparotomy with resection of the diverticulum or segmental resection of small bowel. Non‐surgical management, with intravenous fluids, antibiotics, and bowel rest, has shown to be useful in acute uncomplicated cases. Knowledge of the possibility of conservative management of these stable patients is of great importance, as not all patients with jejunal diverticulitis require a laparotomy.^1^ With a disease prevalent amongst an elderly population, conservative management of acute uncomplicated cases could reduce the morbidity and mortality associated with a large surgery.

Table 1 summarizes recent literature of jejunal diverticulitis case reports and details the demographics, presenting symptoms, imaging modality used, LOS, and management. The average age of presentation from all reviewed case reports, including the patient in our case report, was 70 years with a female to male ratio of 1:4. Abdominal pain was the most common presenting symptom. A CT scan was obtained in each case report, whether as the first imaging modality, or to follow‐up findings seen on an acute series or ultrasound. There is a large range in LOS between the summarized studies, but it appears that non‐operative management has an overall shorter LOS. There was a wide variety in reported LOS between cases presented in Table 1, likely driven by various patients' underlying comorbidities, treatment approaches, and responses to therapy.

**TABLE 1:** Summary of literature regarding management and treatment of jejunal diverticulitis cases

Management	First named author	Year	Demographics	Presentation	Imaging technique	LOS (days)	Treatment
Operative	Aliyeva^5^	2020	64 year, F	LLQ abdominal wall abscess 2/2 enterocutaneous fistula	CT scan	NA	Robotic SBR
	Harbi^6^	2021	40 year, M	RLQ pain, fever	CT scan	NA	Open SBR
	Prough^7^	2019	65 year, M	LLQ pain, nausea, fever	CT scan	5	Open SBR
	Staszewicz^8^	2008	88 year, M	RLQ pain	CT scan	12	Open SBR
	Franca^9^	2010	75 year, M	LLQ pain, rebound tenderness	US, CT scan	6	Unknown approach, SBR
	Gurala^1^	2019	76 year, F	Epigastric pain, confusion, nausea, vomiting, anorexia	Acute Series, CT scan	6	Laparoscopic SBR
	Vayzband^10^	2021	71 year, M	LUQ pain, fever, rigors	CT scan	3	Open SBR
	Leigh^2^	2020	59 year, F	Generalized abdominal pain, nausea, fever	CT scan	7	Open SBR
	Saberski^11^	2012	85 year, M	RLQ pain, nausea, vomiting	Acute Series, CT scan	8	Diagnostic Laparoscopy
	Carmo^12^	2021	76 year, M	Abdominal pain, fever, tachycardia	CT scan x2	17	Laparoscopic SBR
Non‐operative	Matli^13^	2022	41 year, M	Epigastric pain	Acute Series, CT scan	2	IV Piperacillin/Tazobactam *(Abx duration: NA)*
	Samuel^14^	2018	68 year, M	Acute abdominal pain and distention	CT scan	2	IV Ciprofloxacin and Flagyl *(Abx duration: NA)*
	Alam^15^	2014	40 year, M	LLQ pain	US, CT scan	3	IV Ceftriaxone *(Abx duration: NA)*
	Alam^15^	2014	70 year, M	Left flank pain, fever, vomiting	CT scan	NA	IV Ceftriaxone and Flagyl *(Abx duration: NA)*
	Kagolanu^16^	2018	91 year, M	Bilateral flank pain, nausea, vomiting, anorexia	Acute Series, CT scan	NA	IV Ciprofloxacin and Flagyl *(Abx duration: NA)*
	Ejaz^17^	2017	87 year, M	Fever, LLQ pain	Acute Series, CT scan	5	IV Piperacillin/Tazobactam *(Abx duration: 10 days)*
	Ejaz^17^	2017	76 year, M	Post‐prandial abdominal pain, nausea, vomiting	Acute Series, CT scan	2	IV Ciprofloxacin and Flagyl *(Abx duration: 14 days)*
	Dungan^18^	2021	64 year, F	Epigastric pain, constipation, fever, anorexia	CT scan	NA	IV Piperacillin/Tazobactam *(Abx duration: 14 days)*
	Levack^19^	2014	77 year, M	RLQ pain	CT scan	5	IV Ampicillin, Ciprofloxacin, and Flagyl *(Abx duration: 14 days)*

Abbreviations: Abx, antibiotics; CT, computerized tomography; F, female; IV, intravenous; LLQ, left lower quadrant; M, male; NA, not available; RLQ, right lower quadrant; SBR, small bowel resection; US, ultrasound.

## 5 CONCLUSION

In summary, jejunal diverticulitis is an uncommon disease that can cause serious morbidity and mortality. A review of the literature demonstrates a mix of operative and non‐operative management for this disease. The reported case details the progression of symptoms leading to surgical intervention. Early initiation of treatment with intravenous antibiotics could potentially prevent a morbid surgical procedure in the elderly population, where jejunal diverticulitis is most often seen.

## AUTHOR CONTRIBUTIONS

D. Scheese conceived the idea for the document and contributed to writing and editing of the manuscript. Y. Alwatari contributed to writing and editing of the manuscript. J. Khan contributed to writing and editing of the manuscript. A. Slaughter reviewed and edited the manuscript. All authors read and approved the final manuscript.

## FUNDING INFORMATION

None.

## CONFLICT OF INTEREST

None.

## ETHICAL APPROVAL

Ethical approval was not required and patient identifying knowledge was not presented in the report.

## CONSENT

Published with written consent from the patient.

## Data Availability

None.
